# Supplementary material for: Changes in the Milk Metabolome of the Giant Panda (Ailuropoda melanoleuca) with Time after Birth – Three Phases in Early Lactation and Progressive Individual Differences
Source: PLoS One. 2015 Dec 2;10(12):e0143417. doi: 10.1371/journal.pone.0143417 (PMC4668050; doi:10.1371/journal.pone.0143417)
Supplement: S1 File — (PDF) [file pone.0143417.s001.pdf]

Changes in the milk metabolome of the giant panda (*Ailuropoda melanoleuca*) with time after birth – Three phases in early lactation and progressive individual differences

Tong Zhang[1], Rong Zhang[1,2], Liang Zhang[3], Zhihe Zhang[3], Rong Hou[3], Hairui Wang[3], I. Kati Loeffler[3#], David G. Watson[1], Malcolm W. Kennedy[4]*

[1]Strathclyde Institute of Pharmacy and Biomedical Sciences, 161, Cathedral Street, Glasgow G4 0RE, Scotland, UK,

[2]Institute of Clinical Pharmacology, Guangzhou University of Chinese Medicine, No. 12 Jichang Road, Guangzhou 510405, P.R. China,

[3]Sichuan Key Laboratory of Conservation Biology for Endangered Wildlife, Chengdu Research Base of Giant Panda Breeding, 1375 Panda Road, Northern Suburb, Chengdu, Sichuan Province 610081, P.R. China,

[4]Institute of Biodiversity, Animal Health and Comparative Medicine, College of Medical, Veterinary, and Life Sciences, Graham Kerr Building, University of Glasgow, Glasgow G12 8QQ, Scotland, UK

\# Current address: International Fund for Animal Welfare, 290 Summer St., Yarmouth Port, MA 02675, USA

*Corresponding author (MWK)

E-mail: Tong Zhang, tong.zhang.101@strath.ac.uk (metabolomics) or Malcolm Kennedy, malcolm.kennedy@glasgow.ac.uk (biology)

Supporting Information

Table A. Giant panda mother information and dates of milk sample collection.

Table B. MS data of the significant components in giant panda milk selected by multivariate statistical analysis.

Table C. LC-HRMS features verified by comparison with the retention times of authentic standards.

Table D. Compounds detected in the milk samples of giant pandas YY and XYT 20 days postpartum, and which differentiated the two individuals.

Figure A. Body weights of three giant panda cubs over their first 60 days after birth.

Figure B.1. OPLS-DA score plot of 55 giant panda milk samples before and after 7 days of lactation.

Figure B.2. Statistical validation of the OPLS-DA model by permutation analysis.

Figure C. Relative abundance of lactose in the milk samples of the three giant pandas with time postpartum.

Figure D.1. Extracted ion chromatograph of 3' and 6'-Sialyllactose and their MS/MS spectra.

Figure D.2. Extracted ion chromatograph of Gc2-3Lac and its MS/MS and MS/MS/MS spectra.

Figure E.1. OPLS-DA score plot of YY and XYT milk samples 20 days postpartum.

Figure E.2 Statistical validation of the OPLS-DA model by permutation analysis of data from milk samples from giant pandas YY and XYT 20 days after parturition.

Figure E.3. OPLS-DA S-plot of YY and XYT milk samples 20 days postpartum.

Figure F. Statistical validation of the OPLS-DA model by permutation analysis of data from 21 milk samples from giant panda YY versus body weight changes of her cub with time after birth.

Figure G. Relative abundance of isoglobotriose, a common component in the milk of bears, in the milk samples from the three giant pandas with time postpartum .

Figure H. Relative abundance of sn-glycero-3-phosphocholine in the milk samples of the three giant pandas with time postpartum

Figure I. Relative abundance of sn-glycero-3-phosphoethanolamine in the milk samples of the three giant pandas with time postpartum.

Reference for Supporting Information.

Table A. Giant panda mother information and dates of milk sample collection.

Panda mother information	Date of milk collection	Postpartum day
Name: Li Li<br>Studbook No. 387<br>Age: 20 years<br>Parturition date: 28 July 2012<br>Lactation history:<br>12 July 2001, twins. died 19 Sept & 17 Oct, 2001<br>08 July 2002, single, died 08 July 2002<br>03 Aug 2007*, single, died same day<br>19 July 2009, twins<br>24 July 2011, single<br>28 July 2012, twins, 1 died same day<br>Total 17 samples	29 Jul 2012	1
	30 Jul 2012	2
	31 Jul 2012	3
	01 Aug 2012	4
	02 Aug 2012	5
	03 Aug 2012	6
	04 Aug 2012	7
	05 Aug 2012	8
	06 Aug 2012	9
	08 Aug 2012	11
	09 Aug 2012	12
	10 Aug 2012	13
	11 Aug 2012	14
	12 Aug 2012	15
	13 Aug 2012	16
	15 Aug 2012	18
	16 Aug 2012	19
Name: Yuan Yuan<br>Studbook No. 561<br>Parturition date: 25 August 2012<br>Age: 9 years<br>Lactation history:<br>06 Sept 2008, single<br>15 Aug 2010, twins<br>25 Aug 2012, twins, 1 died same day<br>Total 21 samples	26 Aug 2012	1
	27 Aug 2012	2
	28 Aug 2012	3
	29 Aug 2012	4
	01 Sep 2012	7
	04 Sep 2012	10
	05 Sep 2012	11
	08 Sep 2012	14
	09 Sep 2012	15
	10 Sep 2012	16
	13 Sep 2012	19
	16 Sep 2012	22
	17 Sep 2012	23
	19 Sep 2012	25
	20 Sep 2012	26
	21 Sep 2012	27
	23 Sep 2012	29
	24 Sep 2012	30
	25 Sep 2012	31
	01 Oct 2012	37
	10 Oct 2012	46
Name: Xiao YaTou<br>Studbook No. 635<br>Age: 6 years<br>Parturition date: 14 August 2012<br>Lactation history: this was the first parturition<br>17 samples	16 Aug 2012	2
	24 Aug 2012	10
	25 Aug 2012	11
	26 Aug 2012	12
	27 Aug 2012	13
	30 Aug 2012	16
	03 Sep 2012	20
	13 Sep 2012	30
	14 Sep 2012	31

Panda mother information	Date of milk collection	Postpartum day
	17 Sep 2012	34
	18 Sep 2012	35
	19 Sep 2012	36
	20 Sep 2012	37
	21 Sep 2012	38
	30 Sep 2012	47
	01 Oct 2012	48
	09 Oct 2012	56

Table B. MS data for the components in giant panda milk selected as being significant by multivariate statistical analysis. According to the Metabolomics Standards Initiative (MSI) identification levels, all the metabolites listed were designated as level 2 (identified by MS/MS). Compounds are listed in no particular order. Rt, LC retention time. Metlin, the metabolite database, https://**metlin**.scripps.edu/.

Polarity	MZMine ID	MS m/z	Rt (min)	Chemical formula	Compound	MS/MS	MS/MS/MS	Comment
N	1250	172.0620	7.89	C7H11NO4	N-Acetyl-L-glutamate 5-semialdehyde or isomer	128 C6H10NO2		
P	164	232.1540	9.27	C11H21NO4	O-Butanoylcarnitine	173 C8H13O4; 144 C7H14NO2; 85 C4H5O2; 60 C3H10N		Interpreted (144 signature fragment of carnitine)
N	279	155.0100	10.25	C5H4N2O4	Orotate	111 C4H3N2O2		Matched standard spectrum in Metlin
P	158	218.1390	10.30	C10H19NO4	O-Propanoylcarnitine	159 C7H11O4; 144 C7H14NO2; 85 C4H5O2; 60 C3H10N		Interpreted (144 signature fragment of carnitine)
N	501	144.0300	10.71	C5H7NO4	2-Oxoglutaramate	126 C5H5NO3		
P	600	188.1280	12.56	C9H17NO3	???	144 C8H18NO; 114 C5H8NO2; 70 C4H8N		
P	144	174.0870	14.76	C6H11N3O3	5-Guanidino-2-oxopentanoate	146 C5H12N3O2; 130 C5H12N3O; 114 C5H8NO2; 112 C5H10N3; 70 C4H8N		Interpreted (112 signature fragment of guanidine)
N	262	632.2049	14.84	C23H39NO19	3'-Sialyllactose	572 C21H34NO17; 536 C21H30NO15; 470 C17H28NO14; 408 C16H26NO11; 290 C11H16NO8; 170 C7H8NO4	272 C11H14NO7; 194 C9H8NO4; 170 C7H8NO4; 138 C7H8NO2; 98 C5H8NO	Matched MS/MS spectrum in published data

Polarity	MZMine ID	MS m/z	Rt (min)	Chemical formula	Compound	MS/MS	MS/MS/MS	Comment
N	256	318.0960	15.02	C9H22NO9P	HCO_3^- adduct of glycero-3-phosphocholine	171 C3H8O6P		
P	137	258.1100	15.02	C8H20NO6P	sn-glycero-3-Phosphocholine	184 C9H12O4; 104 C5H14ON		Matched standard spectrum in Metlin
N	3050	778.2622527	15.39	C29H49NO23	6'-Sialyl-3-fucosyllactose	650 C19H38O24; 562 C14H28NO22; 470 C17H28NO14; 290 C11H16NO8		Interpreted
N	351	632.2049068	15.62	C23H39NO19	6'-Sialyllactose	572 C21H34NO17; 554 C21H32NO16; 512 C19H30NO15; 470 C17H28NO14; 410 C15H24NO12; 380 C14H22NO11; 308 C11H18NO9; 290 C11H16NO8; 170 C7H8NO4		Matched MS/MS spectrum in published data
N	293	648.1997	15.89	C23H39NO20	Neu5Gc2-3Lac	588 C21H34NO18; 552 C21H30NO16; 424 C16H26NO12; 306 C11H16NO9; 186 C7H8NO5	288 C11H14NO8; 186 C7H8NO5; 158 C6H8NO4; 154 C7H8NO3; 154 C7H8NO3; 116 C4H6NO3	Interpreted (additional oxygen in sialyl group at MS/MS and MS/MS/MS level)
N	266	214.0490	16.08	C5H14NO6P	sn-glycero-3-Phosphoethanolamine	153 C3H6O5P; 140 C2H7NO4P		Interpreted
P	145	216.0630	16.08	C5H14NO6P	sn-glycero-3-Phosphoethanolamine	173 C3H10O6P; 155 C3H8O5P; 62 C2H8NO		Interpreted

Polarity	MZMine ID	MS m/z	Rt (min)	Chemical formula	Compound	MS/MS	MS/MS/MS	Comment
N	263	341.1095	16.75	C12H22O11	Lactose	179 C6H11O6; 161 C6H9O5; 143 C6H7O4; 113 C5H5O3; 101 C4H5O3; 97 C5H5O2; 89 C3H5O3; 87 C3H3O3; 83 C4H3O2		Matched standard spectrum in Metlin
N	258	503.1620	17.77	C18H32O16	isoglobotriose	443 C16H27O14; 425 C16H25O13; 341 C12H21O11; 179 C6H11O6; 161 C6H9O5		Interpreted
N	344	320.0628	20.78	C11H15NO10	beta-Citryl-L-glutamic acid	284 C11H10NO8; 240 C10H10NO6; 128 C5H6NO3; 110 C5H4NO2		Interpreted (128 signature fragment of glutamate)

Table C. LC-HRMS features verified by comparison with the retention times of authentic compound standards (mass tolerance window ± 3 ppm; retention time tolerance window ± 0.5 min). According to the Metabolomics Standards Initiative (MSI), all the metabolites listed here were identified as level 1, indicating that retention times matched with authentic standards**.** Rt = LC retention time.

Polarity	MZMine ID	MS m/z	Rt (min)	Chemical formula	Compound
N	299	88.0403	15.21	C3H7NO2	L-Alanine
N	589	104.0354	16.16	C3H7NO3	L-Serine
P	32	104.107	21.88	C5H14NO	Choline
P	3520	112.0505	11.71	C4H5N3O	Cytosine
N	112	112.0517	10.14	C4H7N3O	Creatinine
P	5	114.0662	10.14	C4H7N3O	Creatinine
N	531	114.0561	13.27	C5H9NO2	L-Proline
P	150	116.0706	13.26	C5H9NO2	L-Proline
N	641	115.0038	16.58	C4H4O4	Fumarate
N	476	116.0718	12.96	C5H11NO2	L-Valine
P	191	118.0862	12.95	C5H11NO2	L-Valine
N	2719	116.0718	11.73	C5H11NO2	Betaine
P	141	118.0862	11.72	C5H11NO2	Betaine
N	2529	117.0194	15.73	C4H6O4	Succinate
N	669	118.0511	14.82	C4H9NO3	L-Homoserine
P	303	120.0655	14.82	C4H9NO3	L-Homoserine
N	843	121.0408	7.78	C6H6N2O	Nicotinamide
P	300	123.0553	7.80	C6H6N2O	Nicotinamide
N	265	124.0074	15.25	C2H7NO3S	Taurine
P	185	126.0219	15.26	C2H7NO3S	Taurine
N	2036	125.0357	11.82	C5H6N2O2	Imidazole-4-acetate
P	365	127.0502	11.83	C5H6N2O2	Imidazole-4-acetate
N	285	128.0354	10.42	C5H7NO3	5-Oxoproline
N	3249	129.0195	7.93	C5H6O4	Citraconate
N	3513	129.0195	15.99	C5H6O4	Mesaconate
N	3800	130.0510	14.92	C5H9NO3	Cis-4-Hydroxy-D-Proline
N	273	130.0623	15.19	C4H9N3O2	creatine
P	37	132.0767	15.21	C4H9N3O2	creatine
N	389	130.0874	11.29	C6H13NO2	L-Leucine
P	168	132.1019	11.26	C6H13NO2	L-Leucine
N	3842	130.0874	11.74	C6H13NO2	L-isoleucine
P	1042	132.1019	11.73	C6H13NO2	L-isoleucine
N	610	132.0303	15.50	C4H7NO4	L-Aspartate
N	289	133.0144	16.56	C4H6O5	(R)-Malate
N	637	133.0508	8.62	C5H10O4	Deoxyribose
N	1285	140.0120	16.09	C2H8NO4P	Ethanolamine phosphate
P	2754	145.0760	6.26	C9H8N2	2-phenyl limidazole
N	550	145.0620	15.48	C5H10N2O3	L-Glutamine
P	186	147.0764	15.47	C5H10N2O3	L-Glutamine
N	578	147.0301	15.75	C5H8O5	Citramalate
N	1438	148.0439	11.90	C5H11NO2S	L-Methionine

Polarity	MZMine ID	MS m/z	Rt (min)	Chemical formula	Compound
P	369	150.0583	11.89	C5H11NO2S	L-Methionine
P	810	150.1124	9.59	C6H15NO3	Triethanolamine
N	1241	150.0562	7.76	C8H9NO2	4-Hydroxyphenylacetaldoxime
P	3032	152.0706	11.39	C8H9NO2	2-Phenylglycine
N	1503	151.0402	9.86	C8H8O3	4-Hydroxyphenylacetate
N	2086	151.0613	13.30	C5H12O5	Xylitol
N	122	157.0368	14.17	C4H6N4O3	Allantoin
P	1558	162.0549	7.94	C9H7NO2	2-Indolecarboxylicacid
N	6752	162.0232	8.14	C5H9NO3S	Acetylcysteine
N	470	163.0614	12.37	C6H12O5	rhamnose
P	379	166.0863	10.53	C9H11NO2	L-Phenylalanine
N	985	165.0560	5.11	C9H10O3	3-Hydroxy-3-phenylpropionic acid-2
N	1628	165.0559	7.73	C9H10O3	3-Hydroxy-3-phenylpropionic acid-1
N	1834	166.9753	18.13	C3H5O6P	Phosphoenolpyruvate
N	423	167.0212	12.92	C5H4N4O3	Urate
N	5435	167.0351	8.58	C8H8O4	Homogentisate
P	3011	170.0925	14.16	C7H12N3O2	N(pi)-Methyl-L-histidine
N	652	168.9909	15.87	C3H7O6P	DL-Glyceraldehyde 3-phosphate
N	275	173.0093	18.46	C6H6O6	Cis-Aconitate
N	1645	173.1046	26.50	C6H14N4O2	L-Arginine
P	707	175.1189	26.49	C6H14N4O2	L-Arginine
N	2182	174.0562	8.74	C10H9NO2	3-Indole-acetate
N	2971	174.0886	15.93	C6H13N3O3	L-Citrulline
P	332	176.1029	15.95	C6H13N3O3	L-Citrulline
N	2317	175.0250	15.99	C6H8O6	D-Glucuronolactone
N	4684	175.0250	14.78	C6H8O6	D-Glucuronolactone
N	5350	176.0389	7.76	C6H11NO3S	N-Formyl-L-methionine
N	5681	177.0406	10.37	C6H10O6	D-Galactono-1,4-lactone
N	885	178.0512	7.75	C9H9NO3	Hippuric acid-1
N	1466	178.0511	5.12	C9H9NO3	Hippuric acid-2
N	312	181.0720	14.31	C6H14O6	D-Sorbitol
N	1869	184.9859	17.54	C3H7O7P	D(+)2-Phosphoglyceric acid
P	2974	189.1597	22.68	C9H20N2O2	N6,N6,N6-Trimethyl-L-lysine
N	331	188.0356	7.75	C10H7NO3	Kynurenic acid-1
N	714	188.0355	5.21	C10H7NO3	Kynurenic acid-2
P	224	190.0498	7.76	C10H7NO3	Kynurenic acid-1
P	497	190.0498	5.25	C10H7NO3	Kynurenic acid-2
N	3399	188.0569	8.70	C7H11NO5	N-Acetyl-L-glutamate
N	254	191.0200	18.65	C6H8O7	Citrate
N	399	195.0512	14.42	C6H12O7	D-Gluconic acid
N	695	202.1088	11.52	C9H18NO4	O-Acetylcarnitine
P	138	204.1230	11.53	C9H18NO4	O-Acetylcarnitine
N	5996	203.0830	12.14	C11H12N2O2	L-Tryptophan
P	3519	205.0972	12.09	C11H12N2O2	L-Tryptophan
N	1307	204.0669	7.84	C11H11NO3	DL-indole-3-lactic acid
N	3405	213.0174	15.03	C5H11O7P	2-Deoxyribose 5-phosphate

Polarity	MZMine ID	MS m/z	Rt (min)	Chemical formula	Compound
N	591	218.1037	8.82	C9H17NO5	Pantothenate
P	295	220.1179	8.84	C9H17NO5	Pantothenate
P	218	222.0971	12.18	C8H15NO6	N-Acetyl-D-Glucosamine
N	426	220.0830	12.19	C9H19NOS2	N-Acetyl-D-Glucosamine
P	3201	227.1136	16.14	C9H14N4O3	L-Carnosine
N	5535	225.0997	16.15	C9H14N4O3	L-Carnosine
N	1236	229.0122	16.14	C5H11O8P	D-Ribose 5-phosphate
N	1916	229.0122	15.63	C5H11O8P	D-Ribulose 5-phosphate
P	2096	238.0933	11.78	C9H11N5O3	Biopterin
N	2919	236.0793	11.78	C9H11N5O3	Biopterin
P	462	244.0927	12.36	C9H13N3O5	Cytidine
N	1075	242.0787	12.36	C9H13N3O5	Cytidine
N	696	243.0625	10.22	C9H12N2O6	Uridine
P	844	258.1083	11.18	C10H15N3O5	5-methylcytidine
N	4010	257.0783	7.80	C10H14N2O6	3-O-Mthyl uridine
P	1613	259.0923	8.56	C10H14N2O6	5-Methyluridine
N	645	258.0388	15.92	C6H14NO8P	D-Glucosamine 6-Phosphate
P	485	260.0528	16.04	C6H14NO8P	D-Glucosamine 6-Phosphate
N	1052	259.0227	17.40	C6H13O9P	glucose 6 phosphate
N	2950	259.0227	16.53	C6H13O9P	glucose 1 phosphate
P	1271	268.1039	9.46	C10H13N5O4	Adenosine
N	5794	267.0737	11.53	C10H12N4O5	Inosine
N	3288	275.0178	18.27	C6H13O10P	6-Phospho-D-gluconate
N	1911	275.1253	16.21	C11H20N2O6	L-Saccharopine
P	3601	284.0987	13.10	C10H13N5O5	Guanosine
P	3060	302.0633	15.42	C8H16NO9P	N-Acetyl-D-glucosamine 6-phosphate
N	1395	300.0494	15.39	C8H16NO9P	N-Acetyl-D-glucosamine 6-phosphate
N	6618	306.0754	15.25	C10H17N3O6S	Glutathione
P	3836	324.0588	15.76	C9H14N3O8P	CMP
N	3293	322.0450	15.76	C9H14N3O8P	CMP
N	703	323.0291	15.65	C9H13N2O9P	UMP
P	644	348.0701	14.23	C10H14N5O7P	AMP
N	801	346.0563	14.23	C10H14N5O7P	AMP
N	3280	347.0403	16.00	C10H13N4O8P	IMP
P	1932	364.0650	17.24	C10H14N5O8P	GMP
N	1972	362.0514	17.25	C10H14N5O8P	GMP
P	163	377.1453	9.03	C17H20N4O6	Riboflavin
N	384	375.1316	9.02	C17H20N4O6	Riboflavin
N	4970	514.2848	7.76	C19H37N11O6	taurocholate
N	3544	606.0753	15.75	C17H27N3O17P2	UDP-N-acetyl-D-glucosamine
N	1737	784.1497	11.95	C27H33N9O15P2	FAD

Table D. Compounds detected in the milk samples of giant pandas YY and XYT 20 days postpartum, and that differentiated the two individuals. The LC-MS features are sorted in the order of their retention times (Rt). Italicised names indicate non-proton adducts and complex ions identified by MZMine 2.10, and confirmed by manually checking the raw LC-HRMS data. The metabolites were annotated based on the Metabolomics Standards Initiative (MSI) identification levels. Level 1: retention times matched with authentic standards (labelled as ST), level 2: identified by MS/MS (labelled as MS), level 3: accurate mass, level 4 unknown. The identified metabolites at levels 1 and 2 were also labelled with the CID codes for the PubChem database.

**YY samples**						
**Polarity**	**MZMine ID**	**MS m/z**	**Rt (min)**	**Chemical formula**	**Compound**	**Identification level**
N	338	129.0559	4.96	C6H10O3	(S)-3-Methyl-2-oxopentanoic acid	3
N	947	203.0023	4.97	C7H8SO5	Hydroxybenzyl alcohol sulfate or isomer	3
N	286	172.9915	5.00	C6H6O4S	Phenol sulfate	3
N	985	165.0560	5.11	C9H10O3	3-(3-Hydroxy-phenyl)-propanoic acid	3
N	4244	109.0295	6.06		*In-source fragment of Sulfacatechol -SO3*	
P	3139	225.0539	6.09			
N	5061	190.9824	6.10		*S34 isotope of Sulfocatechol*	
N	5049	188.9866	6.11	C6H6O5S	3-Sulfocatechol	3
N	362	218.9972	8.52	C7H8SO6	Dihydroxy benzyl alcohol sulfate or isomer	3
N	291	255.0892	9.03	C13H12N4O2	???	4
N	481	90.02773	9.69	C3H6O3	Lactate	3
N	501	144.0304	10.71	C5H7NO4	2-Oxoglutaramate	
N	1259	126.0197	10.72	C5H5NO3	2,3,6-Trihydroxypyridine	3
P	150	116.0706	13.26	C5H9NO2	L-ProlineST	1 CID: 145742
P	194	157.0971	13.27		*ACN adduct of Proline*	
N	311	308.0992	13.81	C11H19NO9	N-Acetylneuraminate	3
P	144	174.0873	14.76	C6H11N3O3	5-Guanidino-2-oxopentanoate	3
N	360	172.0730	14.76	C6H11N3O3	5-Guanidino-2-oxopentanoate	3
N	3039	649.2202	18.32	C24H42O20	Oligosaccharide	4
P	32	104.1070	21.88	C5H14NO	CholineST	1 CID: 305

XYT samples						
**Polarity**	**MZMine ID**	**MS m/z**	**Rt (min)**	**Chemical formula**	**Compound Name**	**Identification level**
N	837	299.2596	4.13	C18H36O3	[FA hydroxy(18:0)] 2S-hydroxy-octadecanoic acid	3
P	156	786.6010	4.27	C44H84NO8P	[PC (18:1/18:1)] 1-(9Z-octadecenoyl)-2-(9Z-octadecenoyl)-sn-glycero-3-phosphocholine	3
P	151	758.5695	4.31	C42H80NO8P	[PC (16:0/18:2)] 1-hexadecanoyl-2-(9Z,12Z-octadecadienoyl)-sn-glycero-3-phosphocholine	3
P	177	246.1698	8.48	C12H23NO4	N-(octanoyl)-L-homoserine	3
P	351	142.0862	8.68	C7H11NO2	L-Hypoglycin	3
P	164	232.1542	9.27	C11H21NO4	O-Butanoylcarnitine^{MS}	2 CID: 439829
N	580	123.9888	9.83			
P	158	218.1386	10.30	C10H19NO4	O-Propanoylcarnitine^{MS}	2 CID: 188824
N	438	124.9915	10.89	C2H6O4S	2-Hydroxyethanesulfonate	3
P	180	121.0720	11.07	C2H8O2N4	Urea dimer	3
P	1015	117.1022	11.39	C5H12N2O	5-Aminopentanamide	4
P	138	204.1230	11.53	C9H18NO4	O-AcetylcarnitineST	1 CID: 18230
P	154	651.2453	14.81		*Ammonium adduct of 3'-Sialyllactose*	
N	262	632.2049	14.84	C23H39NO19	3'-Sialyllactose	
N	265	124.0074	15.25	C2H7NO3S	TaurineST	1 CID: 1123
N	292	249.0224	15.26		*Dimer of Taurine*	
P	146	143.0485	15.26			
N	293	648.1997	15.89	C23H39NO20	Oligosaccharide	4
N	258	503.1624	17.77	C18H32O16	Isoglobotriose^{MS}	2 CID: 11249135
P	142	522.2028	17.78		*Ammonium adduct of isoglobotriose*	
N	1268	834.7859	18.39		*Doubly charged oligosaccharide*	

Figure A. Body weights of three giant panda cubs over their first 60 days after birth. These cubs were the offspring of the pandas whose milk was sampled for this study. Cubs were nursed by their mothers and were supplemented daily with artificial milk formula, per standard husbandry practice at this facility. Therefore, growth rates will have been derived from this dual nutrition and do not necessarily reflect natural giant panda cub growth rates. The graphs of body weight changes with time (left panel) and percent changes from day to day (right panel) indicate that all three cubs underwent the drop in body weight immediately after birth that is typical in mammals. Yuan Yuan's cub, which was used for the exploration of the use of OPLS-DA in inferring components of milk that associate with the rate of cub growth (see Results and Discussion and Fig 3 of the main text), is seen to follow a similar growth pattern to those of the other two cubs fed and treated similarly. There are no comparable data on growth rates of cubs in the wild.

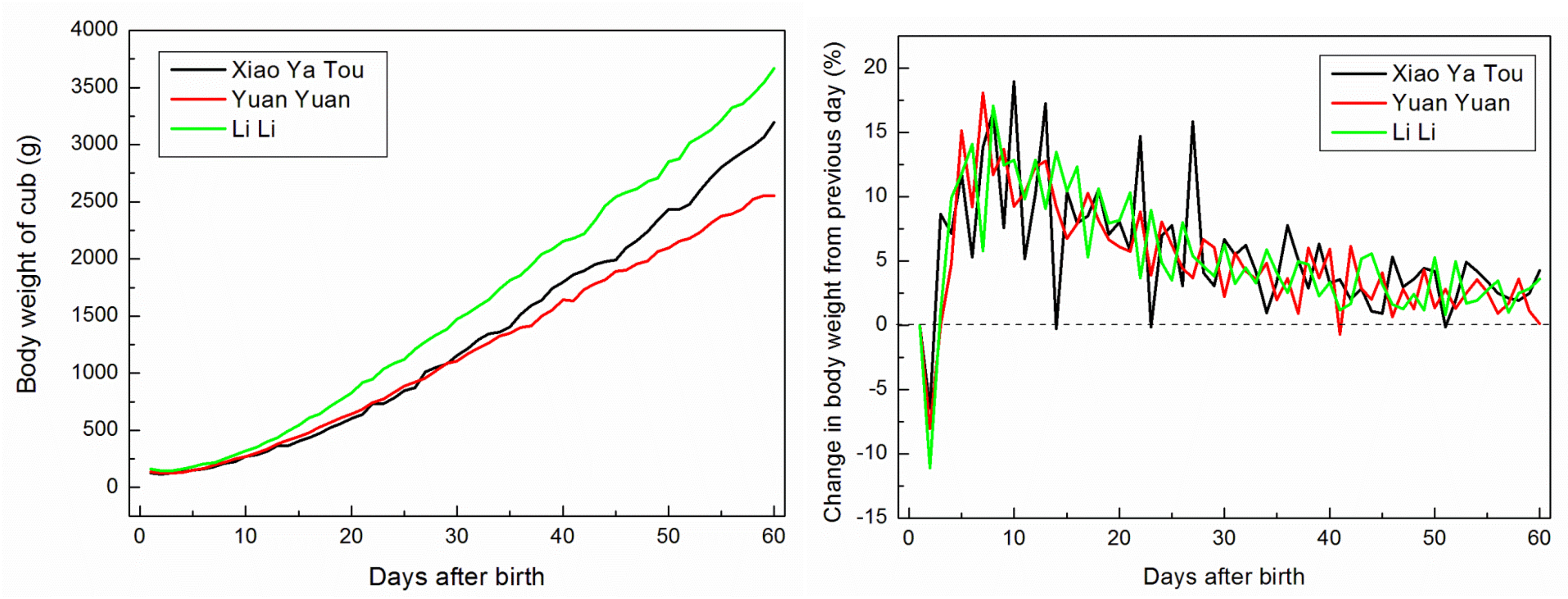

Figure B.1. OPLS-DA score plot of 55 giant panda milk samples before and after 7 days of lactation. Data obtained up to 6 days inclusive after parturition, followed by the period beginning at 7 days post-partum are indicated in red and blue, respectively. The x- and y-axes represent scores for predictive and orthogonal component, respectively, expressed in arbitrary units. OPLS-DA R2X(cum) = 89.9% and Q2(cum) = 80.6%. In OPLS-DA, R2Y (cum) and Q2 (cum) parameters are used for the evaluation of the models, indicating the fitness and prediction ability, respectively. Q2 (cum) > 50% shows that the mode is useful; Q2 (cum) > 90%, is considered excellent.

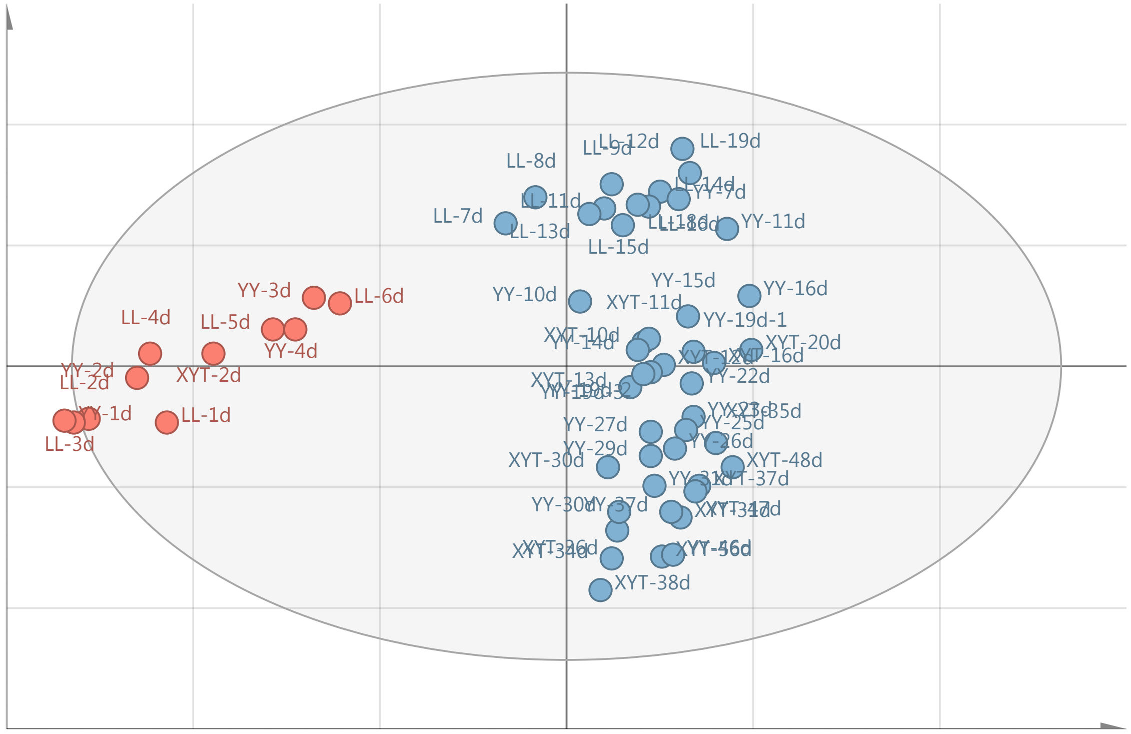

Figure B.2. Statistical validation of the OPLS-DA model by permutation analysis using 57 different model permutations of 55 giant panda milk samples before and after 7 dayspost-partum. The goodness of fit (R2) and predictive capability (Q2) of the original model are indicated to the right and are higher than those of the permuted models to the left. OPLS-DA, orthogonal partial least squares discriminant analysis. Intercepts: R2 = (0.0, 0.439), Q2 = (0.0, -0.473).

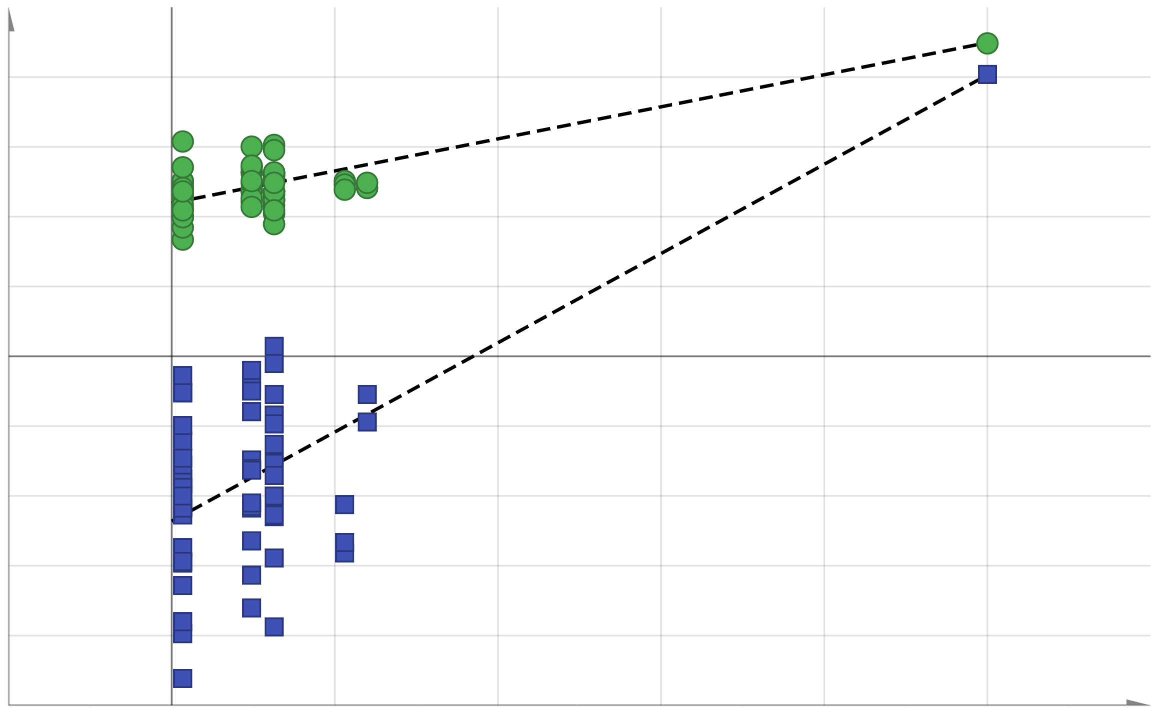

Figure C. Relative abundance of lactose in the milk samples of the three giant pandas with time postpartum, as measured by HILIC-HRMS ($C_{12}H_{22}O_{11}$, retention time (Rt): 16.7min, MZMine ID: N263). Also included are the values for the three artificial milk substitutes analyzed in parallel. The data on lactose in the panda milk samples are also given in ref. (1) but included below are the relative values for the artificial formulae, which were considerably higher than in natural panda milk.

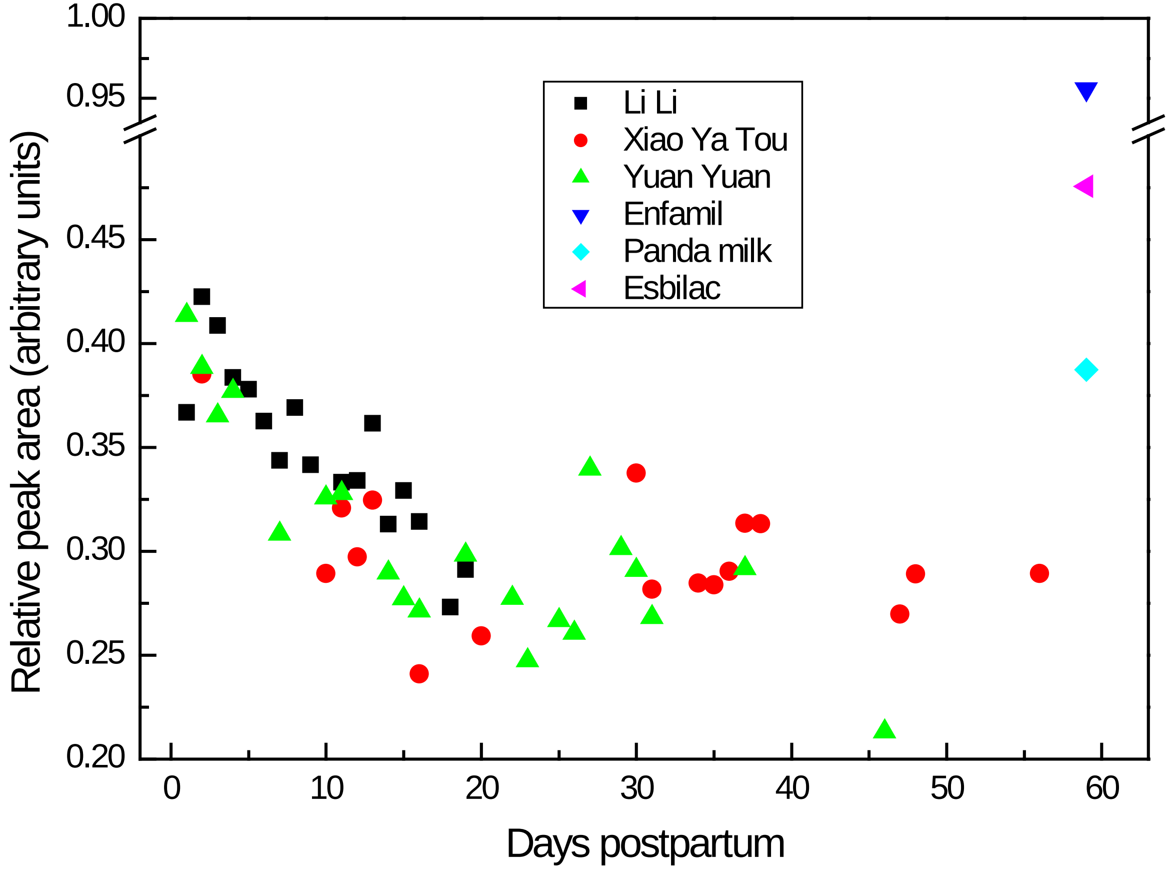

Figure D.1. Extracted ion chromatographs of 3’ and 6’-Sialyllactose and their MS/MS spectra.

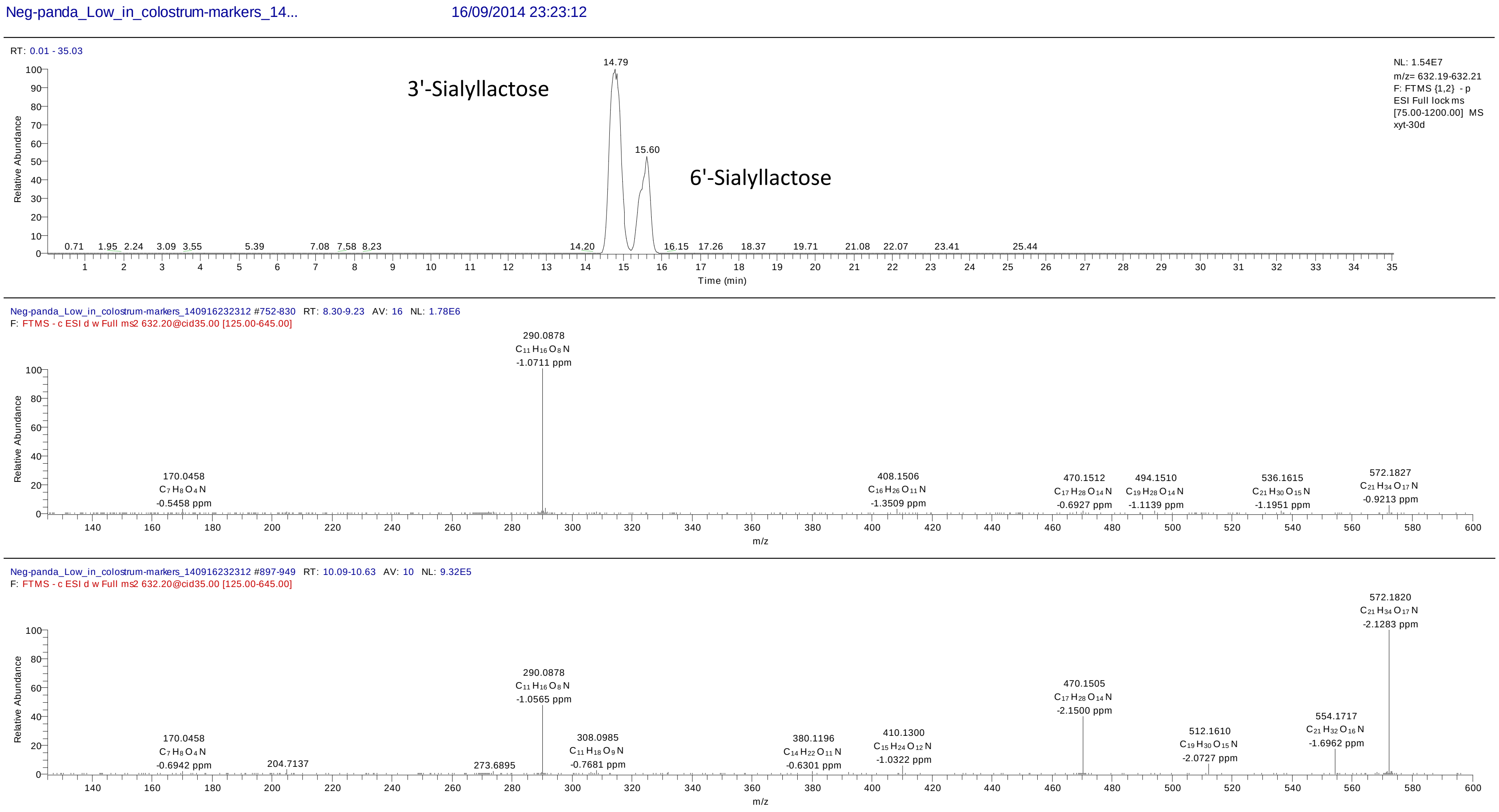

Figure D.2. Extracted ion chromatographs of Gc2-3Lac and its MS/MS and MS/MS/MS spectra.

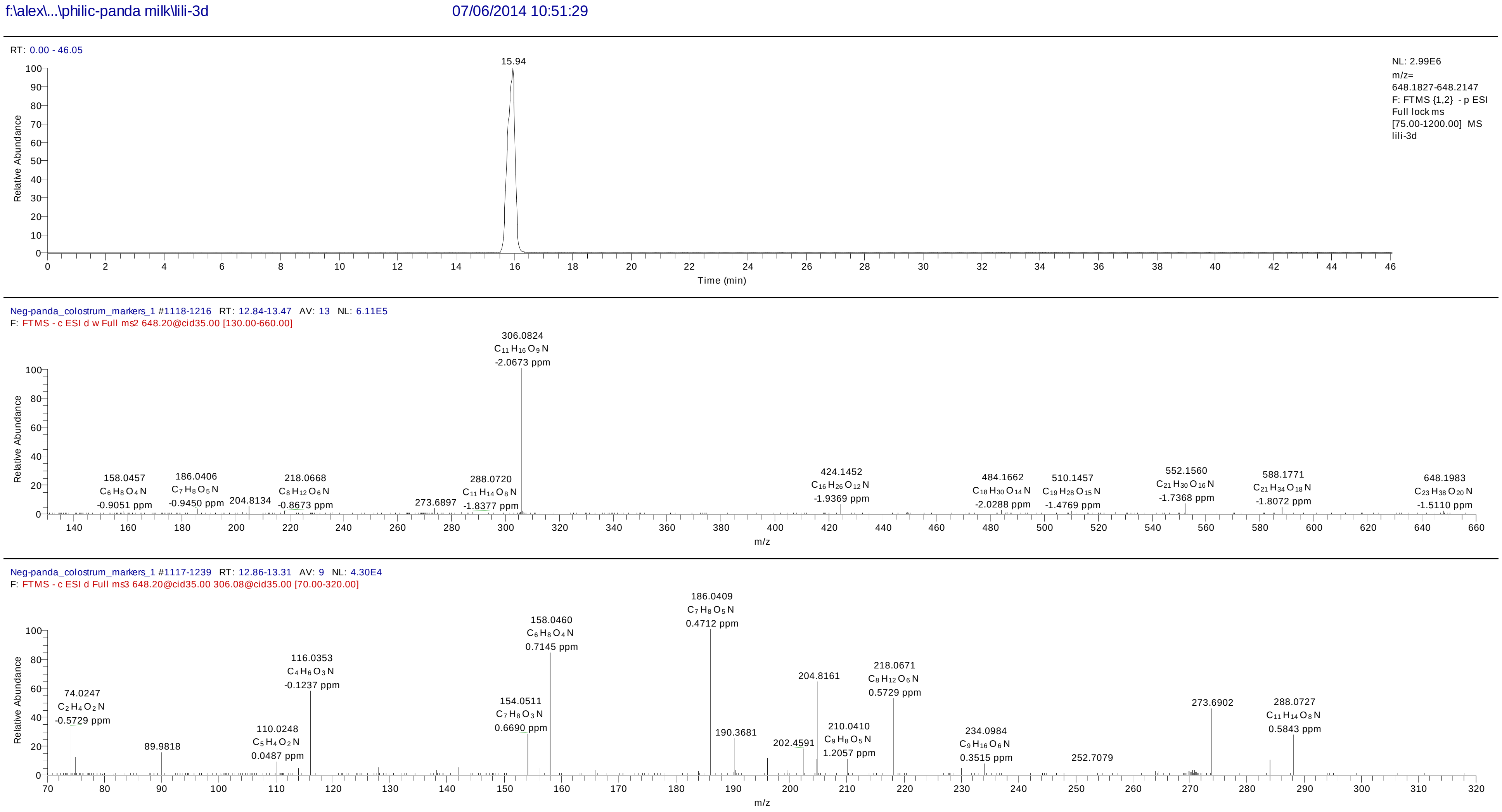

Figure E.1. OPLS-DA score plot of YY and XYT milk samples 20 days postpartum. Data for Xiao Ya Tou (XYT) and Yuan Yuan (YY) are shown in yellow and green, respectively. The x- and y-axes represent scores for predictive and orthogonal component, respectively, expressed in arbitrary units. OPLS-DA: R2X (cum) = 99% and Q2 (cum) = 94.3%.

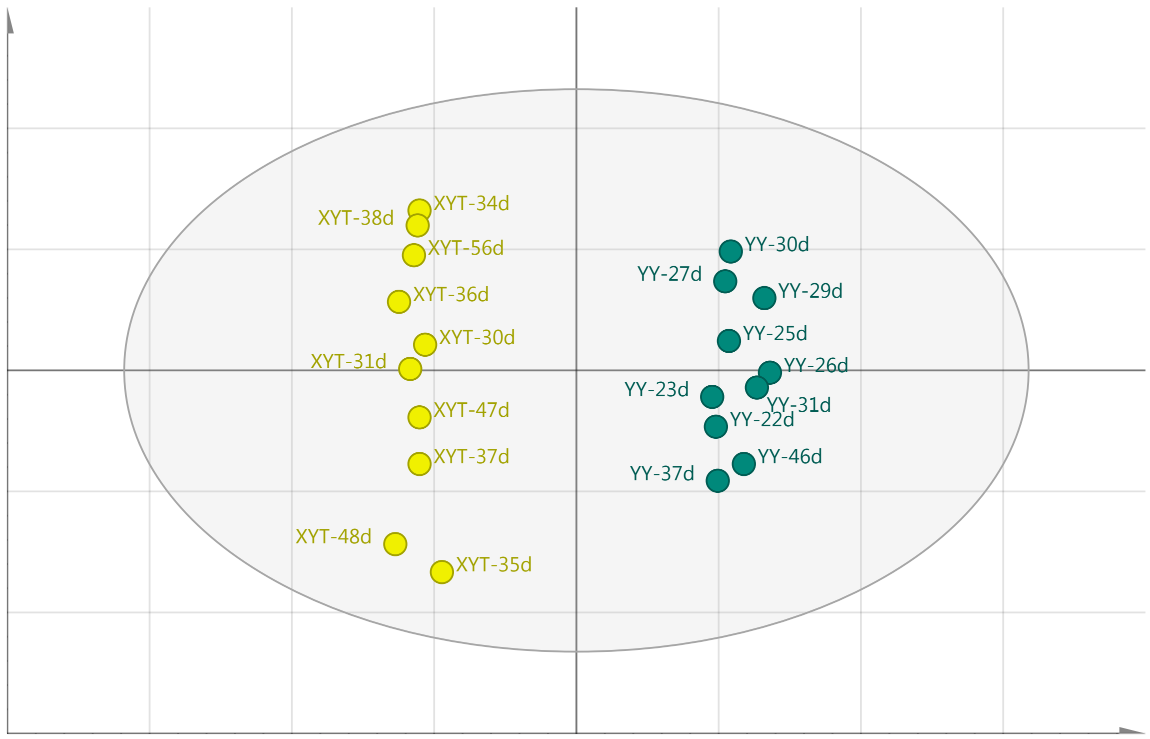

Figure E.2. Statistical validation of the OPLS-DA model by permutation analysis of data from milk samples from giant pandas YY and XYT 20 days after parturition, 20 different model permutations. The goodness of fit (R2) and predictive capability (Q2) of the original model are indicated to the right and remain higher than those of the permuted models on the left. OPLS-DA, orthogonal partial least squares discriminant analysis. Intercepts: R2 = (0.0, 0.817), Q2 = (0.0, -0.548)

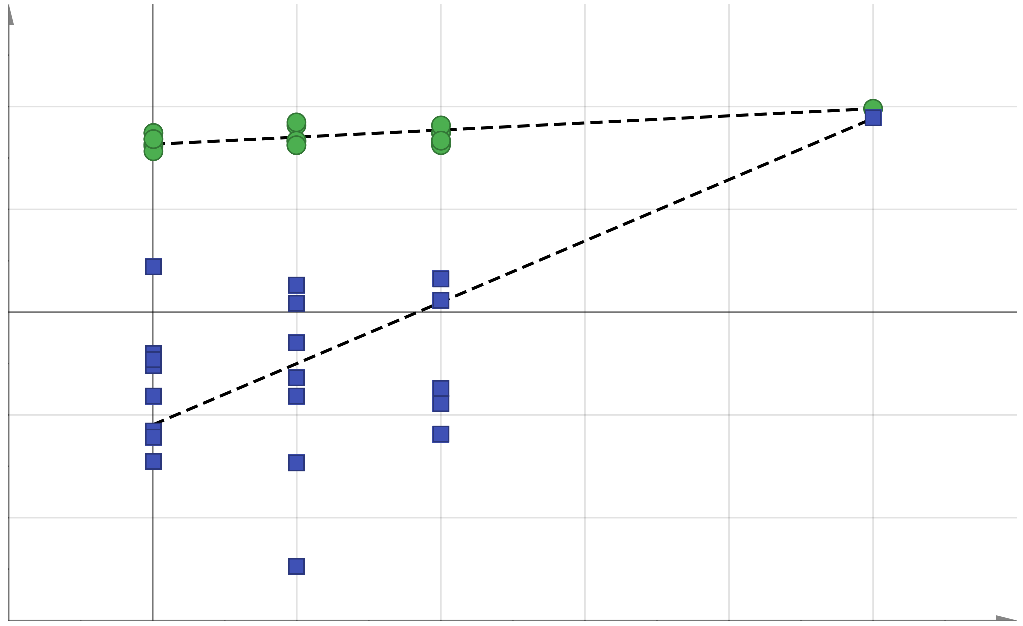

Figure E.3. OPLS-DA S-plot of YY and XYT milk samples 20 days postpartum. The most abundant 20 compounds in the samples of YY and XYT are represented in red and blue, respectively. The HILIC-HRMS data and the identification of these highlighted dots are detailed in Table S4.

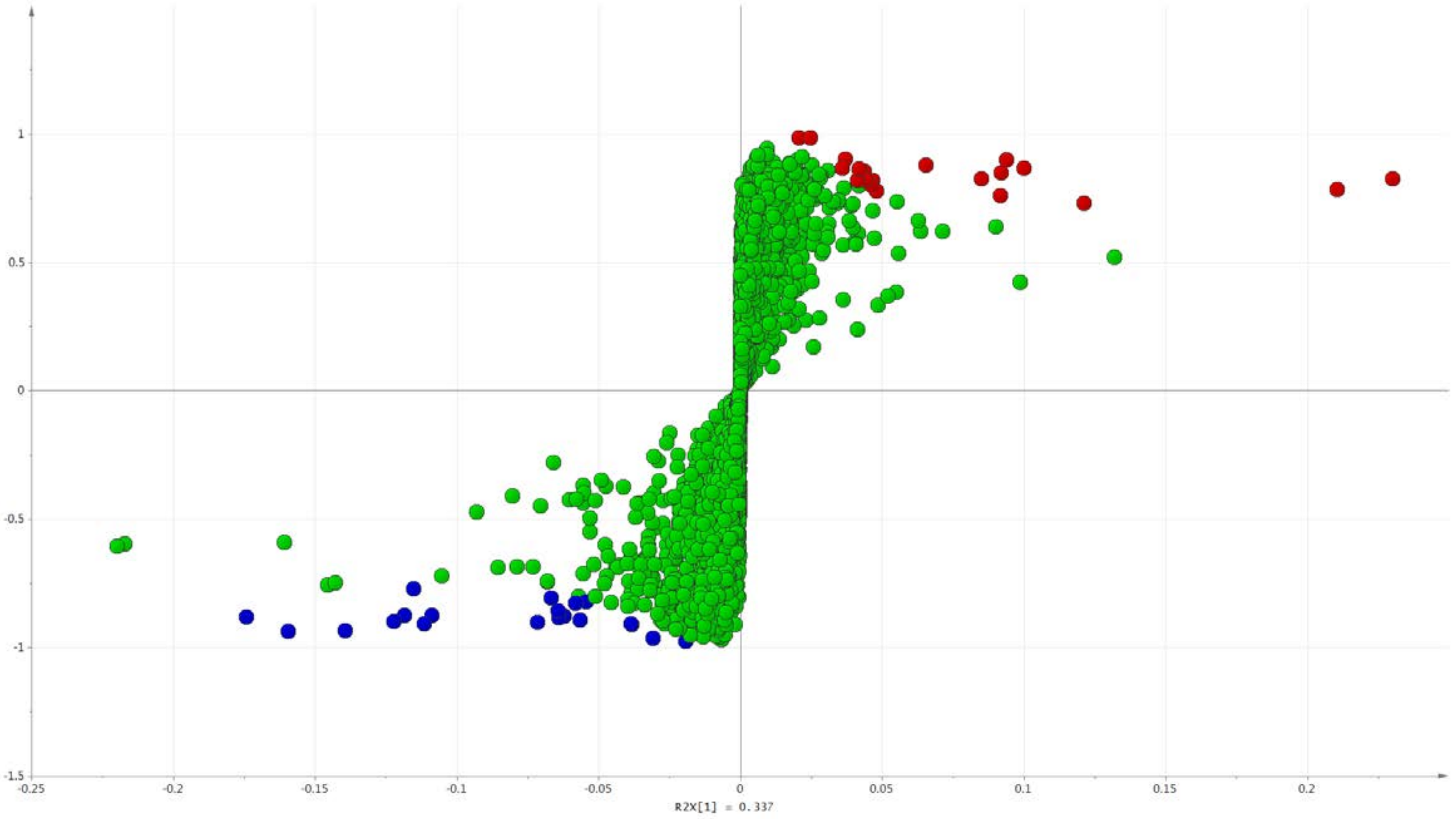

Figure F. Statistical validation of the OPLS-DA model by permutation analysis of data from 21 milk samples from giant panda YY versus body weight changes of her cub with time after birth. Twenty different model permutations are shown. The goodness of fit (R2) and predictive capability (Q2) of the original model are indicated to the right and remain higher than those of the permuted models to the left. OPLS-DA, Orthogonal Partial Least Squares Discriminant Analysis, intercepts: R2 = (0.0, 0.953), Q2 = (0.0, -0.701).

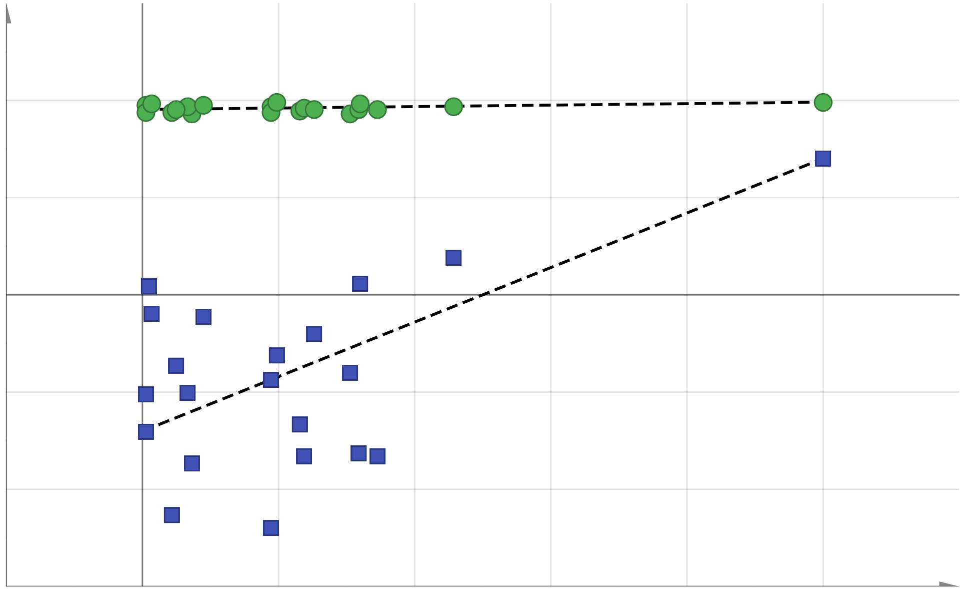

Figure G. Relative abundance of isoglobotriose, a common component in the milk of bears, in the milk samples from the three giant pandas with time postpartum, as measured by HILIC-HRMS ($C_{18}H_{32}O_{16}$, retention time: 17.8min, MZMine ID: N258). Also included are the values for the three artificial milk substitutes analyzed in parallel.

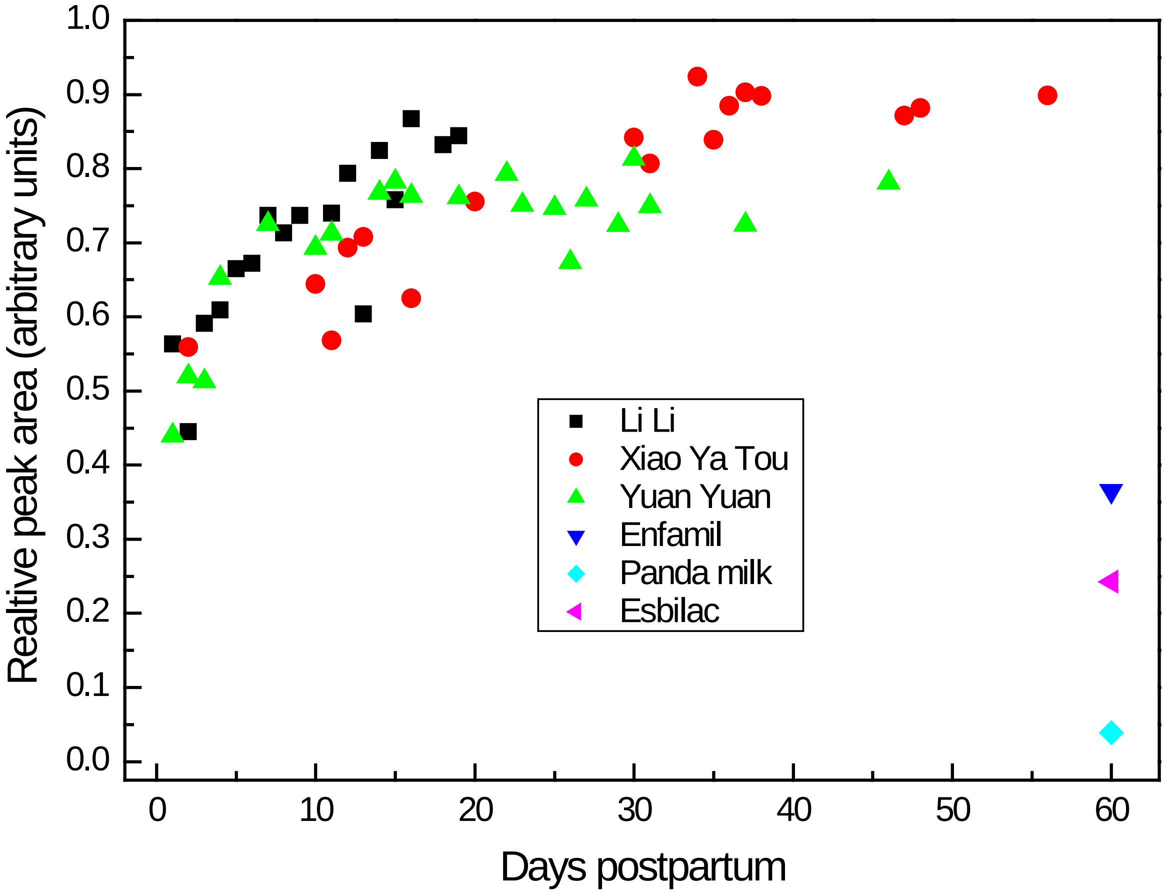

Figure H. Relative abundance of sn-glycero-3-phosphocholine in the milk samples of the three giant pandas with time postpartum, as measured by HILIC-HRMS (C8H20NO6P, retention time: 15.02min, MZMine ID: P137). Also included are the values for the three artificial milk substitutes analyzed in parallel. Peak area values in arbitrary units.

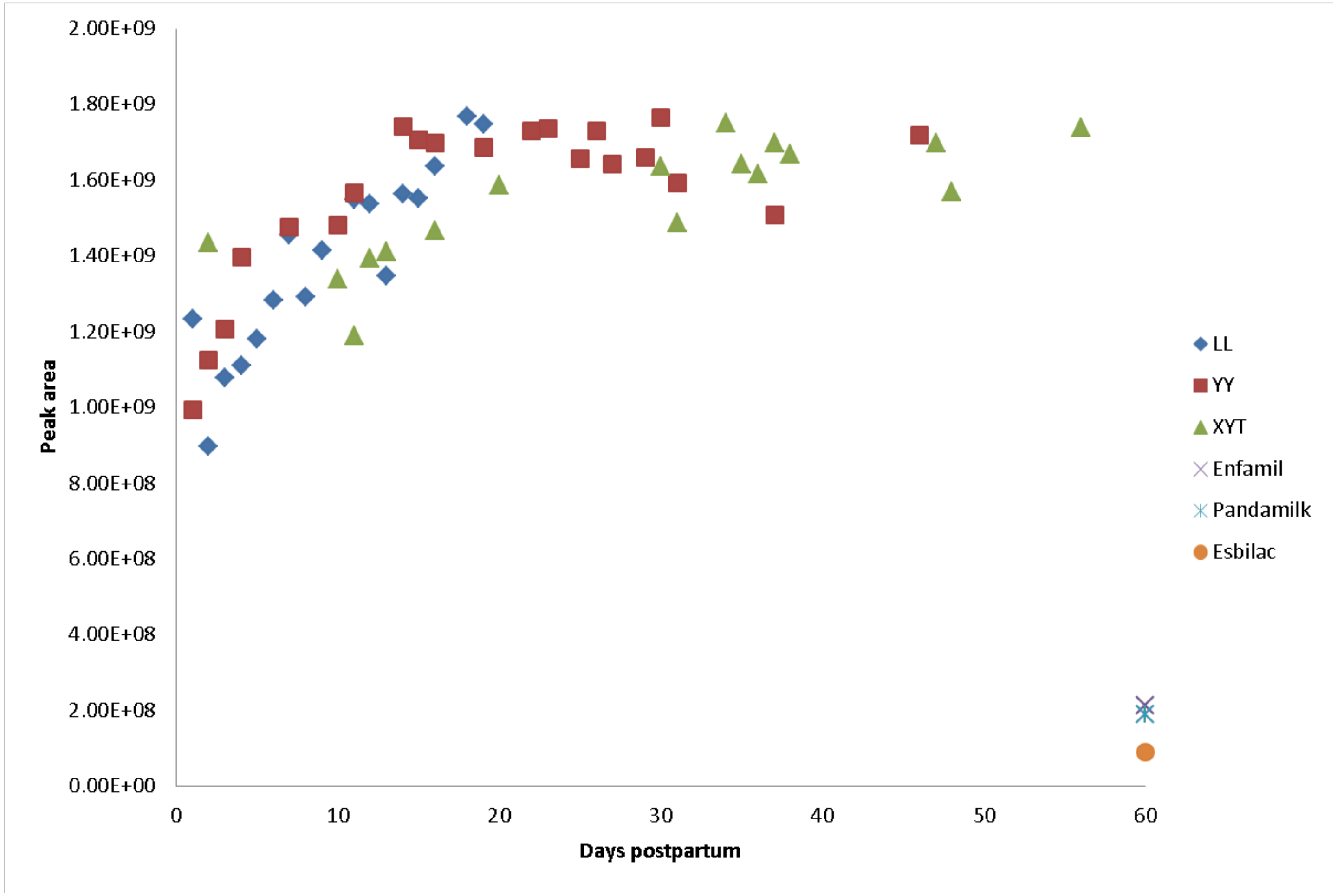

Figure I. Relative abundance of sn-glycero-3-phosphoethanolamine in the milk samples of the three giant pandas with time postpartum, as measured by HILIC-HRMS (C5H14NO6P, retention time: 16.08min, MZMine ID: P145). Also included are the values for the three artificial milk substitutes analysed in parallel. Peak area values in arbitrary units.

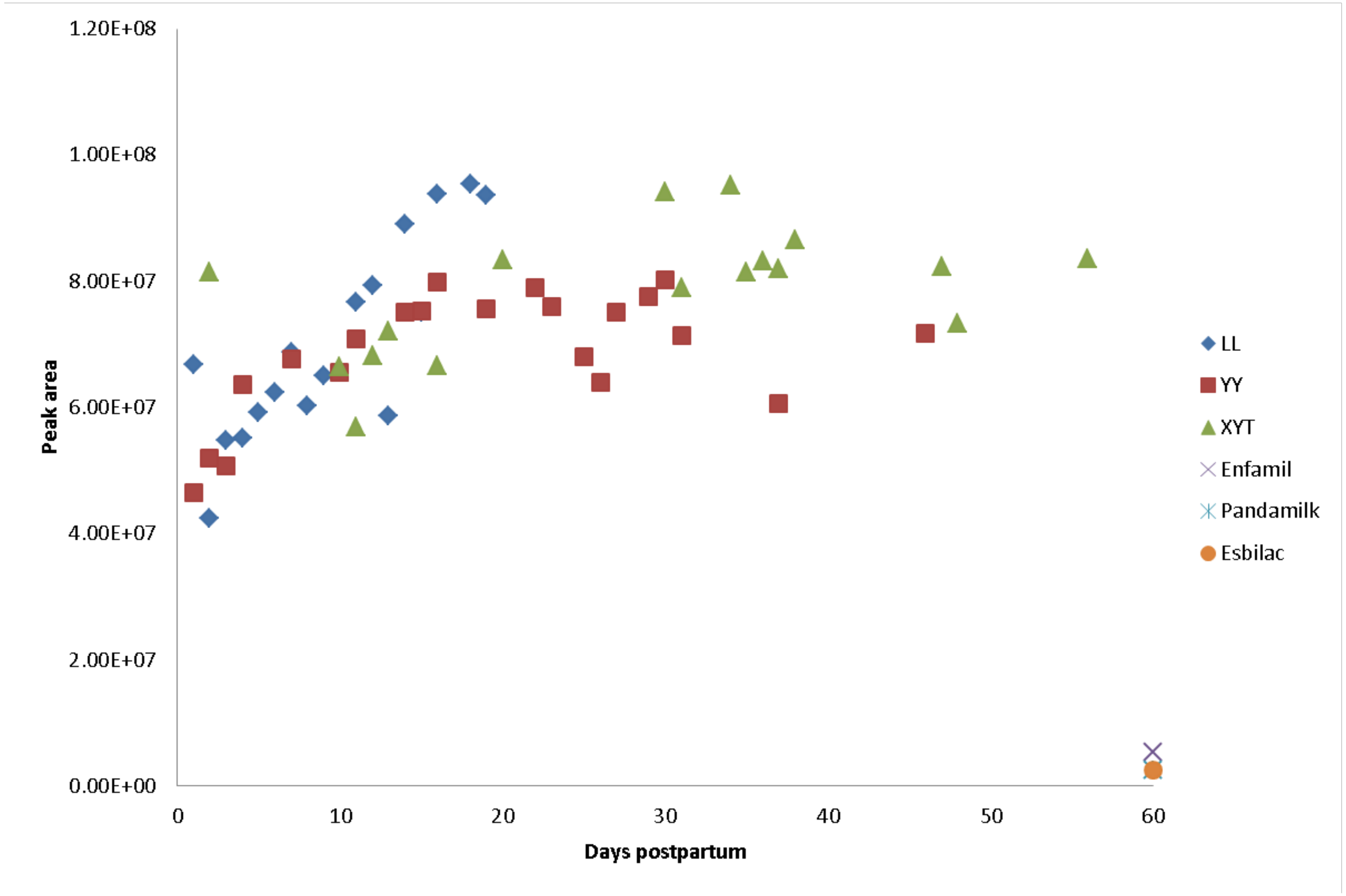

Reference

1. Griffiths K, Hou R, Wang H, Zhang Z, Zhang L, Zhang T, Watson DG, Burchmore RJS, Loeffler IK, Kennedy MW. 2015 Prolonged transition time between colostrum and mature milk in a bear, the giant panda, Ailuropoda melanoleuca. *Royal Society Open Science* **2 (**10). (doi: 10.1098/rsos.150395)
